# High levels of IGF-1 predict difficult intubation of patients with acromegaly

**DOI:** 10.1007/s12020-017-1338-x

**Published:** 2017-06-15

**Authors:** Yu Zhang, Xiaopeng Guo, Lijian Pei, Zhuhua Zhang, Gang Tan, Bing Xing

**Affiliations:** 1 Department of Anesthesiology, Peking Union Medical College Hospital, Chinese Academy of Medical Sciences & Peking Union Medical College, No. 1 Shuaifuyuan, Dongcheng District, Beijing, 100730 People’s Republic of China; 2 Department of Neurosurgery, Peking Union Medical College Hospital, Chinese Academy of Medical Sciences & Peking Union Medical College, No. 1 Shuaifuyuan, Dongcheng District, Beijing, 100730 People’s Republic of China; 3 China Pituitary Disease Registry Center, Peking Union Medical College Hospital, Chinese Academy of Medical Sciences & Peking Union Medical College, No. 1 Shuaifuyuan, Dongcheng District, Beijing, 100730 People’s Republic of China; 4 Department of Radiography, Peking Union Medical College Hospital, Chinese Academy of Medical Sciences & Peking Union Medical College, No. 1 Shuaifuyuan, Dongcheng District, Beijing, 100730 People’s Republic of China

**Keywords:** Difficult intubation, Apnea/hypopnea index, Anesthesia induction, Acromegaly, Upper airway

## Abstract

**Purpose:**

To investigate the characteristics of difficult intubation and identify novel efficient predictors in patients with acromegaly.

**Methods:**

Patients with either untreated acromegaly or non-functional pituitary adenomas were enrolled. Patients with acromegaly underwent hormone assays, upper airway computed tomography and magnetic resonance imaging examinations and preoperative overnight polysomnography. The modified Mallampati classification, mouth opening, neck circumference, and neck extension were assessed, and the Cormack-Lehane grades and the time of tracheal intubation were recorded.

**Results:**

Patients with acromegaly had a higher incidence of difficult intubation (62.5%). The time of tracheal intubation was prolonged, the neck circumference was enlarged, and the neck extension was confined. In patients with acromegaly and difficult intubation, the insulin-like growth factor 1 levels and apnea/hypoxia index were significantly higher compared to patients without difficult intubation (1115.40 ± 253.73 vs. 791.67 ± 206.62 ng/ml, *P* = 0.020; 22.17 ± 23.25 vs. 2.47 ± 2.84, *P* = 0.026, respectively). The bilateral regression analysis revealed that high levels of insulin-like growth factor 1 were an independent risk factor for developing difficult intubation (*p* = 0.042, Exp B = 1.006). The modified Mallampati classification was positively correlated with apnea/hypoxia index and could be calculated using the following logarithmic equation: MMC = 0.2982 * ln (AHI) + 2.1836.

**Conclusions:**

In patients with acromegaly, neck movement is confined, the time of tracheal intubation is prolonged, and the neck circumference is enlarged, and these patients suffer from an increased incidence of difficult intubation (62.5%) during anesthesia induction. The apnea/hypoxia index and insulin-like growth factor 1 levels are both increased in acromegalic patients with difficult intubation, and elevated insulin-like growth factor 1 levels are an independent risk factor of difficult intubation in acromegalic patients.

## Introduction

Elevated levels of growth hormone (GH) and insulin-like growth factor 1 (IGF-1) in patients with acromegaly lead to bone alterations and soft palate hypertrophy. In the respiratory system, acromegalic changes include macroglossia, thickened soft palate and uvula, extended mandible, pharyngeal tissue hypertrophy, around-glottis soft tissue edema, cervical vertebra hyperostosis, and neck movement disorders. Additionally, lesions in these tissues can contribute to upper respiratory tract collapse and stenosis [1–4].

More than 95% of the patients with acromegaly harbor a GH-secreting pituitary adenoma in the sella region, and complete pituitary tumor resection via transsphenoidal surgery is the first-line therapy [5, 6]. Anesthesia and surgical safety play important roles in ensuring a high cure rate and low mortality. Airway management is the most essential and critical aspect of general anesthesia, and tracheal intubation during the anesthesia procedure is the crux of airway management [7]. Because of the upper airway alterations in patients with acromegaly, difficult intubation (DI) was reported to be approximately three times more common than in other surgery patients. In addition, this phenomenon prolongs the time of tracheal intubation (TTI), lowers the success rate of tracheal intubation and increases the incidence of pulmonary infection and long-term mortality [4, 8, 9]. The most effective and efficient method to resolve tracheal intubation difficulty in patients with acromegaly is to recognize and try to prevent DI in advance. To the best of our knowledge, although prediction methods have been reported in the literature, there still exists no consensus in predicting DI in patients with acromegaly. Although the modified Mallampati classification (MMC) is the most widely used method to predict DI during the preoperative period and the upper lip bite test and electronic laryngoscopy can assist in the prediction, there is still an underestimation of more than 10% of patients with acromegaly [8, 10–14]. To investigate the intubation characteristics in patients with acromegaly during general anesthesia, we compared the anesthesia-related indexes between patients with GH-secreting pituitary adenoma and non-functional pituitary adenoma. We also aimed to explore new efficient predictors to help predict DI in patients with acromegaly by comparing the pituitary-related hormone levels, polysomnography results, upper airway radiological changes, and the tracheal intubation characteristics of patients with acromegaly either with or without DI.

### Subjects and methods

#### Study population

Patients with acromegaly who were admitted to the Department of Neurosurgery at Peking Union Medical College Hospital (PUMCH) were enrolled starting from September 1st, 2015. The enrollment criteria were as follows: (1) endocrine indexes [5, 15]: GH nadir >0.4 ng/ml after 75 g oral glucose load, random GH > 1 ng/ml, and high plasma IGF-1 levels (age-related reference range of IGF-1 levels at PUMCH were defined as follows: 18–20 years, 127–584 ng/ml; 21–25 years, 116–358 ng/ml; 26–30 years, 117–329 ng/ml; 31–35 years, 115–307 ng/ml; 36–40 years, 109–284 ng/ml; 41–45 years, 101–267 ng/ml; 46–50 years, 94–252 ng/ml; 51–55 years, 87–238 ng/ml; 56–60 years, 81–225 ng/ml; and 61–65 years, 75–212 ng/ml); (2) typical manifestations of acromegaly; (3) contrast-enhanced MRI showing pituitary adenoma in the sella turcica [16]; (4) none of the patients received any GH-suppressive therapy at the time of surgery, i.e., no previous history of pituitary surgery, irradiation, or medication administration; (5) other pituitary-related hormones at normal reference levels; and (6) no known history of tracheal intubation, upper airway surgery or respiratory disease. During the same period, patients with non-functional pituitary adenoma were enrolled into the control group. The criteria for these patients were (1) pituitary-related hormones within the reference range; (2) confirmed pituitary adenoma via contrast-enhanced MRI; (3) no prior history of pituitary treatment; and (4) no known history of tracheal intubation, upper airway surgery or respiratory disease.

#### Study design

After the patients were admitted, they underwent medical inquiry, physical examination, collection of medical history, contrast-enhanced sella MRI (Discovery MR750, GE), and hormone assays. The blood pressure of patients with hypertension was controlled to less than 140/90 mmHg, and the blood glucose levels of patients with diabetes mellitus (DM) were controlled to normal levels during a fasting period and within 2 h after eating. A solid mass presenting as hypointense or isointense on T1-weighted imaging, hyperintense, or isointense on T2-weighted imaging and reduced reinforcement indicated a pituitary adenoma. The measured pituitary-related hormones included GH, IGF-1, tri-iodothyronine (T3), thyroxine (T4), free tri-iodothyronine (FT3), free thyroxine (FT4), thyroid-stimulating hormone (TSH), adrenocorticotropic hormone (ACTH), cortisol (F), testosterone (T) and prolactin (PRL). Blood samples were collected at 6 am after an 8-h fasting period, with chemiluminescence assays (Siemens Healthcare Diagnostics Products Ltd., UK) and an IMMULITE 2000 analyzer used to measure the GH and IGF-1 levels and electrochemiluminescence assays (Roche Diagnostics GmbH, Germany) and an ADVIA Centaur XP analyzer (Siemens) used to determine the levels of the other hormones. The GH burden was defined as the product of the GH levels (ng/ml) and the disease duration (DD, months), and the IGF-1 burden was defined as the product of the IGF-1 levels (ng/ml) and DD (months).

#### Upper airway imaging and overnight polysomnography

For each enrolled patient with acromegaly, we performed upper airway computed tomography (CT) and magnetic resonance imaging (MRI) as well as overnight polysomnography. While the patients were lying on a horizontal examination table, CT and MRI were performed at two head positions: the straight head position (the straight line that connects the outer canthus and the upper margin of the auricle perpendicular to the examination table) and the extensive head position (the straight line that connects the apex nasi and upper margin of the auricle perpendicular to the examination table). The CT (Definition Flash, SIEMENS) and MRI (Discovery MR750, GE) scans were not contrast-enhanced, and the scanning range was set from the bottom of the anterior cranial fossa to the sixth cervical vertebra.

Four axial planes were set, including the soft palate level, the uvula level, the tongue base level, and the epiglottis level. The maximum anteroposterior diameter, maximum transverse diameter, cross-sectional area, and thickness of the lateral and the posterior pharyngeal walls were measured on each axial level. The maximum thickness and cross-sectional area of the soft palate as well as the maximum thickness of the uvula were measured on the midsagittal plane.

An 8h overnight polysomnography was performed on patients with acromegaly in the ward by respiratory physicians, and the apnea/hypopnea index (AHI), which indicates the average number of apnea and hypopnea events per hour during sleep, was recorded. Patients were diagnosed with sleep apnea/hypopnea syndrome (SAHS) when they had related symptoms and the AHI exceeded 5 [17].

#### General anesthesia

Anesthetists at PUMCH are required to have a minimum of 5 years of experience before they can independently complete anesthesia induction, tracheal intubation, and anesthesia recovery for patients with acromegaly. Mouth opening, mandible length, thyroid-chin distance, degree of neck extension, and neck circumference were evaluated by the anesthetists during the preoperative period. The MMC was differentiated based on the visualized structures: class I (soft palate, fauces, uvula, tonsillar pillars); class II (soft palate, fauces, uvula); class III (soft palate, base of the uvula); and class IV (soft palate not visible) [18].

During the operative procedure, patients were subjected to standard anesthetic monitoring, including electrocardiography, non-invasive blood pressure, and pulse oxygen saturation (SpO_2_). Then, the preoxygenation was performed with pure oxygen (6 L/min) after establishing the venous pathway. General anesthesia was induced with midazolam (0.2 mg/kg), sufentanil (0.15 µg/kg), propofol (TCI, 6 µg/ml), and rocuronium bromide (0.8 mg/kg). After the loss of bilateral eyelash reflexes, mask ventilation was initiated. After the muscle relaxant took effect, we evaluated the difficulty classification of mask ventilation (grade 1, standard one-handed mask ventilation technique; grade 2, two-handed mask ventilation technique; grade 3, oropharyngeal airway-assisted technique) (TOF = 0) and then performed the tracheal intubation.

Appropriately sized Macintosh laryngoscope blades were used to expose the glottis structures, and the available pharyngeal view was classified according to the Cormack-Lehane grading system [19]. To improve glottis structure exposure in patients with a grade III or IV laryngeal view, external laryngeal pressure from the assistant was allowed. If the TTI exceeded 1 min, the intubation would be stopped, and the preoxygenation was performed to prepare for a second attempt at tracheal intubation. DI was defined as either an MMC of III or IV or a successful tracheal intubation after two or more attempts [10, 11]. The TTI was calculated from the time of the Macintosh laryngoscope blade placement into the patient’s mouth to proper inflation of the endotracheal tube cuff.

Propofol (TCI, 4–5 µg/ml) and a mixture of oxygen (50%) and nitrous oxide (50%) were administered to maintain anesthesia. Sufentanil (0.1 µg/kg) and rocuronium bromide (0.15 mg/kg) were intermittently provided according to the surgical procedure. Tracheal extubation was performed after the patients recovered total consciousness and normal respiration.

#### Statistics

SPSS version 17.0 (SPSS Inc., IBM, USA) was used to analyze the data. The results are shown as the means ± standard deviations (SDs), numbers or percentages. Levene’s test was used to evaluate data distributions; quantitative data with normal distributions were assessed using the *t*-test, whereas variables lacking normal distributions were assessed using the Mann–Whitney U test. The *χ* ^2^ test compared differences of categorical variables. Binary logistic analysis was used to exclude confounding factors and determine independent risk factors for DI. AHI and MMC were illustrated as a scatter diagram with two logarithmic trendlines. Statistical significance was defined as *p* < 0.05.

## Results

### Study population

This study enrolled 16 patients with acromegaly (10 men and 6 women, average age 41 years) and 19 patients with non-functional pituitary adenomas (6 men and 13 women, average age 47 years) as a control group. Compared with the control patients, patients with acromegaly had an increased incidence of DM (37.50 vs. 5.26%, *p* *=* 0.032) and higher levels of blood glucose (6.24 ± 1.45 vs. 4.78 ± 0.44 mmol/L, *p* *=* 0.001), GH (20.49 ± 15.03 vs. 0.45 ± 0.40 ng/ml, *p* < 0.001), IGF-1 (994.00 ± 281.17 vs. 198.26 ± 110.14 ng/ml, *p* < 0.001), GH burden (1653.00 ± 1910.72 vs. 31.67 ± 63.33 ng*m/ml, *p* *=* 0.004) and IGF-1 burden (79,640.25 ± 55,347.61 vs. 8822.21 ± 12,205.21 ng*m/ml, *p* < 0.001). The levels of other pituitary-related hormones in both groups were all within their respective reference ranges and showed no significant differences. Although the patients with acromegaly had increased average height, weight and BMI; prolonged disease duration; and higher incidence of hypertension, no significant differences were observed between these groups (Table 1).

**Table 1:** Demographic characteristics and hormone levels of patients with acromegaly and control patients

	Patients with acromegaly (*n* = 16)	Controls (*n* = 19)	*P*
Male, *n* (%)	10 (62.50%)	6 (31.58%)	0.095
Age (year)	41.25 ± 7.21	47.26 ± 12.81	0.092
Height (cm)	168.75 ± 5.85	164.58 ± 6.61	0.059
Weight (kg)	75.16 ± 14.09	68.24 ± 12.06	0.127
BMI	26.24 ± 3.65	25.15 ± 4.00	0.411
DD (month)	84.00 ± 64.40	45.79 ± 56.51	0.070
HT, *n* (%)	4 (25.00%)	1 (5.26%)	0.156
DM, *n* (%)	6 (37.50%)	1 (5.26%)	0.032
LDT (mm)	17.74 ± 8.91	24.79 ± 13.12	0.078
Fasting glucose (mmol/l)	6.24 ± 1.45	4.78 ± 0.44	0.001
Fasting GH (ng/ml)	20.49 ± 15.03	0.45 ± 0.40	<0.001
GH nadir (ng/ml)	12.49 ± 6.75	NA	NA
GH burden, (ng*m/ml)	1653.00 ± 1910.72	31.67 ± 63.33	0.004
IGF-1 (ng/ml)	994.00 ± 281.17	198.26 ± 110.14	<0.001
IGF-1 burden, (ng*m/ml)	79640.25 ± 55347.61	8822.21 ± 12205.21	<0.001
T3 (ng/ml)	1.03 ± 0.35	0.95 ± 0.22	0.453
T4 (μg/dl)	8.14 ± 0.96	7.54 ± 1.56	0.193
FT3 (pg/ml)	2.90 ± 0.48	2.77 ± 0.98	0.622
FT4 (pg/ml)	1.15 ± 0.28	1.11 ± 0.21	0.624
TSH (m IU/l)	1.35 ± 0.96	2.05 ± 1.40	0.100
ACTH (pg/ml)	26.43 ± 17.40	19.49 ± 11.96	0.189
*F* (μg/dl)	12.20 ± 6.16	10.53 ± 5.20	0.389
*T* (ng/ml)	1.08 ± 0.69	0.74 ± 0.68	0.146
PRL (ng/ml)	15.01 ± 14.58	17.93 ± 12.53	0.528

*BMI* body mass index, *DD* disease duration, *HT* hypertension, *DM* diabetes mellitus, *LDT* largest diameter of tumor

### Anesthetic indexes in patients with acromegaly and the controls

All the patients underwent successful tracheal intubation and transsphenoidal surgery. Anesthetic indexes in patients with acromegaly differed significantly with the controls. In patients with acromegaly, the degree of neck extension was decreased (35.00 ± 6.58 vs. 41.00 ± 5.59, degree, *p* *=* 0.006), the neck circumference was increased (40.41 ± 3.29 vs. 37.58 ± 3.51, cm, *p* *=* 0.020), the total time of the tracheal intubation was prolonged (41.13 ± 28.78 vs. 26.16 ± 7.57, cm, *p* = 0.036), and the percentage with DI was increased (62.50 vs. 15.79%, *p* = 0.006). Eight patients with acromegaly (50.0%) and three controls (15.8%) required external laryngeal pressure. Two patients with acromegaly had a TTI exceeding 1 min; the MMC of these patients were III and IV, respectively. The intubation of both patients succeeded during the second attempt with external laryngeal pressure. The mouth opening, mandible length, and thyroid-chin distance were not different between the groups (Table 2).

**Table 2:** Predictive anesthetic indexes for difficult intubation of patients with acromegaly and control patients

	Patients with acromegaly (*n* = 16)	Controls (*n* = 19)	*P*
Mouth opening (cm)	4.34 ± 0.79	4.37 ± 0.60	0.917
Mandible length (cm)	12.09 ± 1.25	11.50 ± 1.33	0.187
Thyroid-chin distance (cm)	9.36 ± 1.48	8.68 ± 1.42	0.176
Degree of neck extension (°)	35.00 ± 6.58	41.00 ± 5.59	0.006
Neck circumference (cm)	40.41 ± 3.29	37.58 ± 3.51	0.020
Total time of the tracheal intubation (s)	41.13 ± 28.78	26.16 ± 7.57	0.036
Difficult intubation (Mallampati), *n* (%)	10 (62.50%)	3 (15.79%)	0.006
Cormack-Lehane grade of III or IV, *n* (%)	7 (43.75%)	4 (21.05%)	0.273
Mask ventilation difficulty level above 2, *n* (%)	3 (18.75%)	1 (5.26%)	0.312
External laryngeal pressure, *n* (%)	8 (50.00%)	3 (15.79%)	0.065
Intubation after two or more attempts, *n* (%)	2 (12.50%)	0 (0.00%)	0.202

### DI and the correlative factors in patients with acromegaly

There were no significant differences in sex, age, physical examination, disease duration, and past medical history of patients with acromegaly either with or without DI. Although most of the pituitary-related hormones were not different, the fasting IGF-1 levels in patients with acromegaly and DI were significantly higher than those in patients without DI (1115.40 ± 253.73 vs. 791.67 ± 206.62, *p* = 0.020) (Table 3). The binary logistic analysis revealed that elevated IGF-1 levels were an independent risk factor for developing DI in patients with acromegaly (Exp B = 1.006, *p* = 0.042).

**Table 3:** Clinical comparisons between patients with acromegaly with and without DI

	Patients with acromegaly with DI (*n* = 10)	Patients with acromegaly without DI (*n* = 6)	*P*
Male (*n*)	6	4	1.000
Age (year)	42.30 ± 7.42	39.50 ± 7.12	0.471
Height (cm)	168.90 ± 5.20	168.50 ± 7.34	0.900
Weight (kg)	76.65 ± 16.53	72.67 ± 9.56	0.601
BMI	26.66 ± 4.38	25.53 ± 2.13	0.568
DD (month)	86.40 ± 57.70	90.00 ± 79.87	0.784
HT, *n* (%)	4 (40.00%)	0 (0.00%)	0.234
DM, *n* (%)	4 (40.00%)	2 (33.33%)	1.000
LDT (mm)	17.37 ± 10.34	18.37 ± 6.74	0.837
Glu (mmol/l)	6.27 ± 1.49	6.18 ± 1.52	0.912
GH (ng/ml)	20.82 ± 18.50	19.94 ± 7.81	0.914
GH Nadir (ng/ml)	11.89 ± 6.55	13.49 ± 7.59	0.663
GH burden (ng*m/ml)	1683.36 ± 2292.73	1602.40 ± 1218.90	0.938
IGF-1 (ng/ml)	1115.40 ± 253.73	791.67 ± 206.62	0.020
IGF-1 burden (ng*m/ml)	87,051.06 ± 58,553.59	67,288.00 ± 52,294.86	0.508

*BMI* body mass index, *DD* disease duration, *HT* hypertension, *DM* diabetes mellitus, *LDT* largest diameter of tumor, *DI* difficult intubation

Comparisons of the upper airway CT and MRI of patients with acromegaly either with or without DI indicated no significant differences in the respiratory tract diameters, pharyngeal soft tissue, and thickness of the soft palate and uvula regardless of the head positions (Suppl. 1 and 2).

**Fig. 1:**
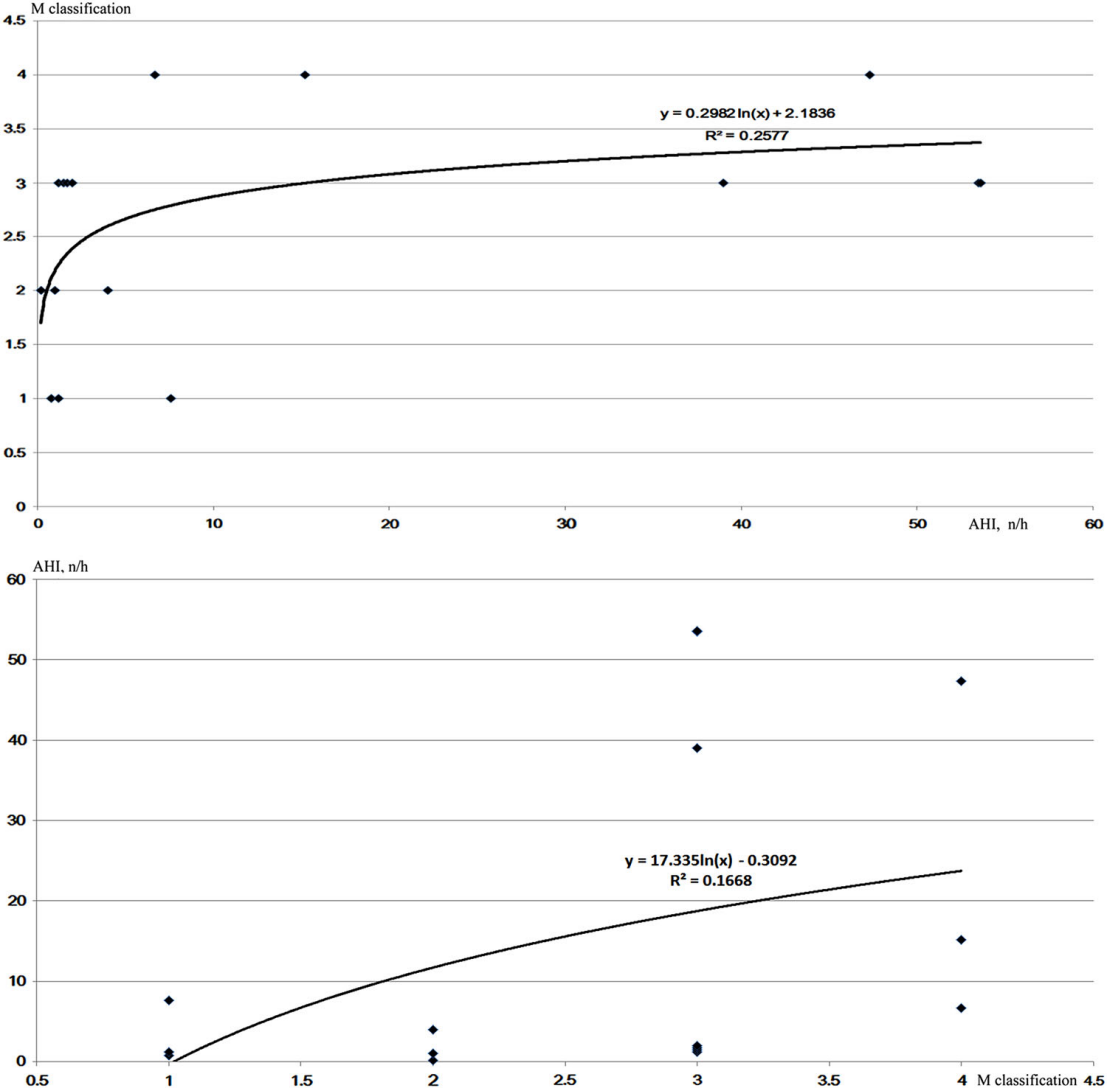
Scatterplot of the relationship between the AHI and the modified Mallampati classification (MMC) scores. The equations and *R* ^2^ values are labeled beside the curves. These two *curves* indicated positive correlations between AHI and the MMC scores

The morbidity of SAHS in patients with acromegaly was 43.8%, which increased to 60% in patients who experienced DI. Patients with acromegaly and DI had more severe SAHS than patients without DI, as indicated by the higher AHI (22.17 ± 23.25 vs. 2.47 ± 2.84, *p* = 0.026) (Table 4). However, the binary logistic analysis revealed no significant difference in the AHI between acromegalic patients with and without DI. We plotted the AHI and the corresponding MMC scores on a scatter plot and found that these two indexes were positively correlated (Fig. 1) and could be expressed using the following logarithmic equations: (1) Mallampati = 0.2982 * ln (AHI) + 2.1836 (*R* ^2^ = 0.2577); and (2) AHI = 17.335 * ln (Mallampati)−0.3092 (*R* ^2^ = 0.1668).

**Table 4:** Sleep monitoring and predictive anesthetic indexes in patients with acromegaly with or without DI

	Patients with acromegaly with DI (*n* = 10)	Patients with acromegaly without DI (*n* = 6)	*P*
AHI	22.17 ± 23.25	2.47 ± 2.84	0.026
Mouth opening (cm)	4.40 ± 0.84	4.25 ± 0.76	0.727
Mandible length (cm)	12.40 ± 1.37	11.58 ± 0.92	0.219
Thyroid-chin distance (cm)	9.78 ± 1.66	8.67 ± 0.82	0.151
Degree of neck extension (°)	36.50 ± 7.47	32.50 ± 4.18	0.192
Neck circumference (cm)	40.80 ± 3.83	39.75 ± 2.27	0.555
Time of the tracheal intubation (s)	45.40 ± 35.24	34.00 ± 12.36	0.462
OSAHS, *n* (%)	6, 60.00%	1, 16.67%	0.145
Mask ventilation difficulty level over 2, *n* (%)	3, 30.00%	0, 0%	0.250
External laryngeal pressure, *n* (%)	7, 70.00%	1, 16.67%	0.119
Intubation with 2 or more attempts, *n* (%)	2, 20.00%	0, 0%	0.500

*AHI* apnea/hypopnea index, *OSAHS* obstructive sleep apnea hypopnea syndrome, *DI* difficult intubation

## Discussion

In this study, we demonstrated that patients with acromegaly had an increased risk of developing DI (62.5%); these patients also presented a prolonged TTI, increased neck circumference, and reduced range of neck extension vs. patients with non-functional pituitary adenomas. In patients with acromegaly and DI, the IGF-1 levels were significantly higher and the AHI was larger compared to patients without DI. Additionally, higher IGF-1 levels were an independent risk factor for developing DI. The upper airway CT and MRI had little effect on predicting DI in patients with acromegaly.

The literature reports that the incidence of DI is increased in patients with acromegaly, ranging from 9 to 26% [7, 11, 12, 20]. Macroglossia and upper respiratory tract stenosis result in poor glottis exposure, which compounds the difficulty of tracheal intubation. Although anesthetic techniques and skull base surgeries have made revolutionary advances, tracheal intubation for patients with acromegaly is still the most difficult and challenging procedure during general anesthesia [9, 20].

DM, secondary obesity and limited head and neck movement have been recognized as risk factors for DI [21]. In 1985, Voyagis et al. [22] investigated the airway in obese patients and stated that the tracheas of individuals with a higher body mass index (BMI) were more difficult to intubate. However, there were also several studies that reported no such connection between BMI and DI [23, 24]. Our results agree with the latter opinion. In our study, the incidence of DM was higher in patients with acromegaly than that in the control patients. Additionally, the percentage of individuals with DI was also higher among patients with acromegaly. This indicated that DM presented as a risk factor for DI in patients with acromegaly. Tumor size did not show an association with the incidence of DI [20], and the present study was consistent with that result.

We found that the angle of neck extension was reduced by 6° and that the neck circumference was increased by 3 cm in patients with acromegaly. Neck hypoactivity made adjusting the head position difficult during anesthesia induction. Thickened fat and soft tissue hypertrophy could lead to increased neck circumference and airway stenosis, both of which increase the difficulty of intubation. The TTI increased by 15 s in patients with acromegaly; this was caused by difficulty in exposing the glottis, which in turn could contribute to increased probability of hypoxemia and other adverse effects related to anesthesia. We noticed a trend that in patients with acromegaly, mask ventilation was more difficult, the percentage of individuals requiring external laryngeal pressure was increased, and successful intubation with two or more attempts was observed in more than 10% of the patients. The above anesthetic characteristics suggested that the intubation risk is greatly increased in patients with acromegaly.

There was no consolidated standard to predict DI. The literature indicates that MMC, the Cormack-Lehane grade, the simplified predictive intubation difficulty score, the upper lip bite test, a tracheal time exceeding 1 min, successful intubation with two or more attempts, a short thyroid-chin distance and confined neck movement were all reported indexes for predicting and evaluating DI [8, 10–14, 20, 25]. A single method to predict whether patients with acromegaly will experience DI preoperatively is difficult and often results in unanticipated DI and anesthesia failure [9]. Thus, identifying a proper method to complement the MMC score in predicting DI is urgent. Because the MMC score is the most widely adopted method to assess DI [11, 18, 20, 26] and has been established as the standard for predicting DI at our center, we used this method to stratify patients with acromegaly and explore correlative factors.

Upper airway CT and MRI can effectively and efficiently evaluate soft tissue hypertrophy and bony alterations that can influence tracheal intubation. These radiological findings were expected to be helpful in evaluating anatomic changes and predicting the difficulty of intubation. Previous studies revealed typical radiological characteristics of the respiratory changes in patients with acromegaly, including macroglossia, an extended mandible, soft palate and uvula hypertrophy, and a thickened pharyngeal wall [6, 20, 27–29]. However, no significant differences in these characteristics were observed in the radiological imaging in this study. This might because of the increased pharyngeal muscular tension when the patients were undergoing upper airway CT and MRI—the positioning and procedure does not properly imitate the condition of pharyngeal muscular tension loss and tracheal collapse during anesthesia induction. Future prospective studies with larger sample sizes may aid in exploring the correlation between radiological changes and DI in patients with acromegaly.

The original dogma stated that GH acts through the direct effects of IGF-1 metabolized from GH in the liver. In recent years, this opinion was replaced by a new standpoint that GH as well as IGF-1, which was metabolized and synthesized from GH, mutually affected the cellular receptors [28, 30–32]. GH prompts cell differentiation, whereas IGF-1 regulates cell cloning and hyperplasia [3, 33]. Schmitt et al. [20] stated that the levels of GH were not associated with DI in patients with acromegaly. Our result was in agreement with this finding and demonstrated that IGF-1 levels played a critical role in the process of pharyngeal hypertrophy and respiratory tract stenosis as well as contributed to a higher incidence of DI in patients with acromegaly.

The prevalence of SAHS in patients with acromegaly (approximately up to 70% [13, 34, 35]) is more common than that among the general population. The process of tracheal intubation in patients with SAHS is more difficult [36]. Lee et al. [37]. demonstrated that the incidence of DI was more common in patients with SAHS and that increased AHI and neck circumference could predict DI in patients with SAHS. The consensus on acromegaly complications published in 2013 recommended that patients with acromegaly should undergo overnight polysomnography performed by respiratory physicians [15].

AHI is the diagnostic index of SAHS that can indicate its severity. The present study showed that 43.8% of patients with acromegaly had SAHS; furthermore, 60% of patients with DI were also diagnosed with SAHS vs. 16.7% of patients without DI. The average AHI levels were approximately ten times greater in patients with DI than those with without DI. According to the logarithmic equation of the AHI and MMC, we could calculate the MMC of patients with acromegaly with AHI and the equation preoperatively and prepare for the tracheal intubation in advance.

Preoperative anesthetic interview is critical for successful anesthesia and surgery. We suggested that anesthetists should predict the difficulty of tracheal intubation and recognize patients with DI prior to surgery based on the IGF-1 levels, AHI, TTI, neck circumference, and range of neck extension; however, the IGF-1 levels were the most critical of the variables. We recommend that each pituitary disease center establish a multidisciplinary team (MDT) comprising neurosurgeons, experienced anesthetists, respiratory physicians, and radiologists. Every patient with acromegaly should undergo the most suitable and comprehensive evaluation before surgery by this MDT.

In conclusion, the incidence of DI in patients with acromegaly during anesthesia induction is higher. The TTI is prolonged, the neck movement is confined and the neck circumference is increased in these patients. The IGF-1 levels and AHI were higher in patients with acromegaly and DI than in acromegaly patients without DI, but upper airway imaging revealed no significant differences between these two groups. Elevated IGF-1 levels are an independent risk factor that can predict an increased likelihood of developing DI in patients with acromegaly.

## Electronic supplementary material

Supplementary Information

Supplementary Table 1

Supplementary Table 2
